# Configurational Fragility of Forest Landscapes Under Multiple Anthropic Uses

**DOI:** 10.1002/ece3.73460

**Published:** 2026-06-11

**Authors:** Jessyca Janyny de Oliveira Saraiva‐Maia, Milena Dutra da Silva, Wallace Beiroz, Nadjacleia Vilar Almeida

**Affiliations:** ^1^ Programa de Pós‐Graduação em Ecologia e Sustentabilidade Universidade Federal da Paraíba Rio Tinto Paraíba Brazil; ^2^ Departamento de Engenharia e Meio Ambiente Universidade Federal da Paraíba Rio Tinto Paraíba Brazil; ^3^ Programa de Pós‐Graduação em Ecologia e Evolução Universidade Estadual de Feira de Santana Feira de Santana Bahia Brazil

**Keywords:** agricultural expansion, biodiversity conservation, forest fragmentation, landscape ecology, savanna formation, tropical watershed, watershed management

## Abstract

The conversion of native vegetation to anthropogenic land uses and landscape fragmentation are primary drivers of biodiversity loss and ecosystem services decline in tropical watersheds. Understanding how land use shapes the spatial configuration of forest fragments is essential to inform conservation and restoration strategies. We evaluated the configurational fragility of forest remains in the Lower São Francisco River Watershed, Brazil, combining structural metrics (area, core area and shape index) with land use and land cover (LULC). Fragility levels were defined through hierarchical classification and their relationship with LULC was tested using distance‐based redundancy analysis (db‐RDA). A principal component analysis (PCA) first summarized LULC variation. The watershed is dominated by anthropogenic matrices, with agriculture and pasture occupying 66.9% of the area, mainly pasture (57.7%). This resulted in 37,022 highly reduced and discontinuous forest fragments, of which 72.2% exhibited high configurational fragility. Savanna formation, the most representative native vegetation, had 96.1% of its patches within intermediate to high fragility levels. The PCA revealed three dominant gradients: herbaceous formations, wooded sandbank vegetation and wetlands (PC1), contrast between savanna formations and pasture (PC2), and heterogeneity of forests, forest plantation and mosaic of uses (PC3). db‐RDA confirmed this pattern (adjusted *R* ^2^ = 0.362), with CAP1 explaining 92.2% of the constrained variation, demonstrating a strong opposition between natural environments and anthropogenic land uses. This study shows that configurational fragility is strongly driven by anthropogenic LULC, revealing that landscapes with the same forest cover may differ in fragility depending on the spatial organization of the remnants. In the area analyzed, sublevels High III and Intermediate I predominated, where small fragments dominated by edges coexist with larger and irregular patches, with occasional occurrences of Low III. As a management guideline, it is recommended to protect strategic remnants, intervene in intermediate areas, and restore zones of high fragility. Future studies should integrate these sublevels with functional indicators and temporal dynamics.

## Graphical abstract

This study revealed that 72.2% of forest fragments and 96.1% of savannas have high configurational fragility, with a predominance of High III and Intermediate I sublevels and occasional occurrences of low fragility (Low III). Fragility is driven by pastures (57.7%), showing that landscapes with the same forest cover differ in fragility depending on the spatial organization of the fragments. It is recommended to protect strategic remnants, intervene in intermediate areas, and restore zones of high fragility to ensure ecological connectivity and ecosystem services.

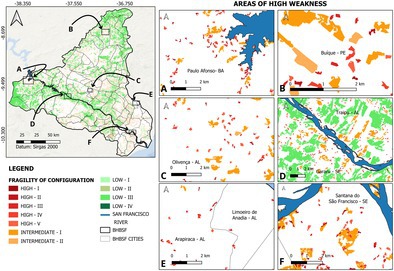

## 1 Introduction

Landscape modification and fragmentation are a global and well‐recognized problem in conservation biology (Cunningham and Lindenmayer 2017; Rivas and Navarro‐Cerrillo 2024). Replacement of native vegetation by anthropic land uses such as pastures and monocultures, particularly soybeans and sugarcane in South America, has been one of the main drivers of landscape simplification, both compositionally, due to the reduction in soil cover diversity, and configurationally, due to the increase in patch size, contributing to the decline of native vegetation (Gámez‐Virués et al. 2015; Mesquita et al. 2025; Song et al. 2021).

In tropical regions, fragmented forest landscapes have become increasingly pronounced over the last two decades of the 21st century (Ma et al. 2023). In Brazil, this scenario has a historical background, especially in watersheds where most of the land matrix is dedicated to agriculture and livestock production (Cabernard et al. 2024; Colman et al. 2024). The predominance of pastures and croplands is a common feature in semi‐arid Brazilian watersheds, and such changes in land use tend to alter both the structural and functional characteristics of natural landscapes at multiple scales (Farias et al. 2023; Silveira et al. 2022).

Watersheds and, consequently, hydrological services are particularly vulnerable to land use changes caused by urbanization and agricultural expansion (Qiu and Turner 2015). Environmental problems often arise in landscapes dominated by anthropogenic environments, such as intensification of erosive processes, river siltation, and loss of ecological functions and ecosystem services provided by vegetation cover and river systems (Bendito et al. 2023; Da Silva et al. 2025; Moura et al. 2023). Vegetation cover plays a key role in hydrological services and biodiversity maintenance, making it critical for environmental management strategies. Studies such as those by Piffer et al. (2021) and Gianuca et al. (2024) clearly demonstrate that maintaining and restoring native vegetation cover is crucial for the ecological and social balance of watersheds under pressure of human activities.

Understanding landscape structure is fundamental for the effective maintenance of ecosystems, since spatial characteristics, such as composition (types of land cover) and configuration (spatial arrangement of land covers), directly influence ecological and socioenvironmental processes in anthropized landscapes (Medupin et al. 2020; Suárez‐Castro et al. 2022). These structural analyses not only reveal spatial patterns but also support the identification of priority areas for conservation and sustainable management.

In this context, the ecological sustainability of fragmented habitats depends both on spatial configuration (size, quality, and connectivity of fragments) and on the intensity of cumulative anthropic disturbances, such as deforestation and climate change (Arroyo‐Rodríguez et al. 2020; Fahrig et al. 2019; Haddad et al. 2015). The interaction between these factors determines the ability of the remnants to preserve biodiversity and ecosystem functions in the long term.

Landscape configuration analyses are widely employed to comprehend the spatial organization of forest remnants in the landscape (Laurance et al. 2018; May et al. 2019; Ribeiro et al. 2009a). These advances have led to the consolidation of understanding regarding the structural condition of patches. The aforementioned understanding indicates that the condition of patches is contingent not only on the total amount of habitat available, but also on the spatial organization of that habitat within the landscape.

In this context, the spatial configuration of patches is not limited to isolated attributes; rather, it constitutes a relational property of the landscape. Landscape metrics, including patch area, core area, and geometric complexity, represent distinct yet structurally interdependent dimensions. When evaluated in isolation, these metrics provide only partial descriptions of the structure. Nevertheless, it is their interaction that defines the effective structural quality of forest remnant patches.

It is in this theoretical framework that classification approaches aimed at the diagnostic synthesis of landscape configuration emerge. Among these approaches, the notion of structural fragility is particularly noteworthy. This notion was introduced by Lopes et al. (2022) and Matias et al. (2020). This perspective sought to establish a connection between the quantitative measurement of spatial structure and the evaluative interpretation of the condition of forest remnants. This objective was accomplished by means of the organization of multiple structural dimensions into hierarchical categories of structural fragility.

However, two conceptual limitations can be identified in the application of the notion of structural fragility. The first of these is the absence of hierarchical subclasses, which have been shown to restrict classification sensitivity by grouping fragments with relevant differences in effective core area availability or edge exposure intensity under broad categories (Rivas et al. 2022). The second issue pertains to the utilization of the term “structural”, which has the potential to engender interpretive ambiguities. Within the domain of ecological literature, the term “structure” is predominantly associated with the organization of biological communities, species composition, or trophic arrangements. The presence of such terminological overlap has the potential to obfuscate the exclusively spatial focus of the approach under consideration in this study.

**FIGURE 1:**
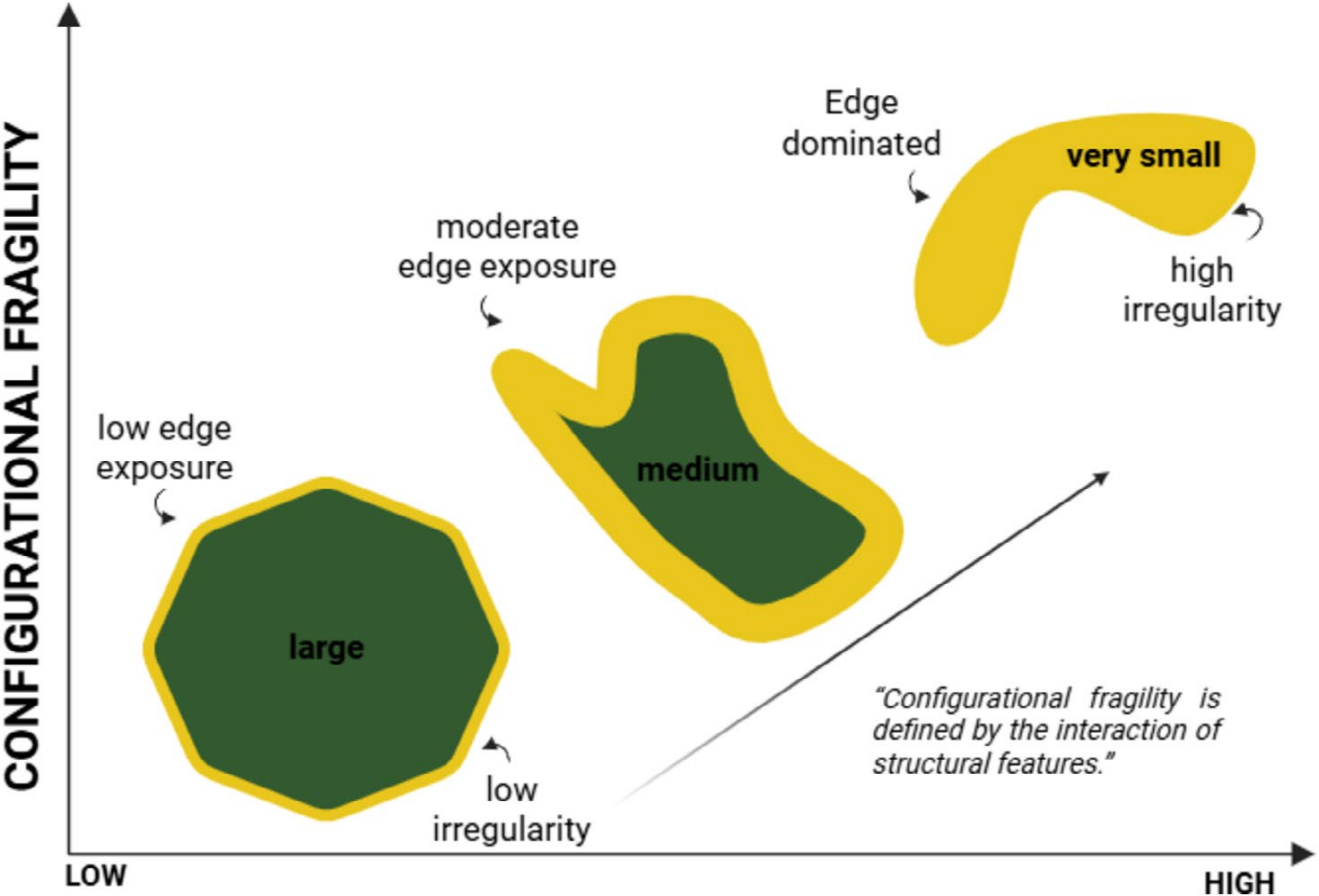
Conceptual diagram of configurational fragility. The yellow areas represent the edges of the forest, and the green areas represent the core of the forest.

In view of the aforementioned issues, we propose the adoption of configurational fragility as a conceptual refinement. This refinement highlights the centrality of spatial configuration and incorporates hierarchical classes capable of capturing internal nuances in landscape organization. By emphasizing the configurational dimension, this concept more precisely delineates its analytical scope and enhances the diagnostic sensitivity of the spatial configuration classification of patches in the landscape. Configurational fragility is defined by the interaction between area, edge exposure, and shape irregularity, forming a continuous gradient with inter‐ and intra‐class nuances. The categories presented represent reference points on this continuum: low (large area, low edge exposure, regular shape); intermediate (medium size, moderate exposure, irregular shape); high (very small area, dominated by edge, very irregular shape) (Figure 1).

The historical anthropization of landscapes to meet the demands of population growth and land speculation has led to the expansion of agricultural, industrial and urban areas. Consequently, questions arise regarding the influence of multiple land uses on the configurational fragility of forest remnants. This study seeks to understand the landscape contexts in which forest remnants are most likely to exhibit high levels of configurational fragility, testing the hypothesis that areas dominated by anthropic land uses present greater fragility than those covered by native formations. Our objective is to identify patterns that can guide restoration and management strategies in tropical watersheds.

## 2 Materials and Methods

### 2.1 Study Area

**FIGURE 2:**
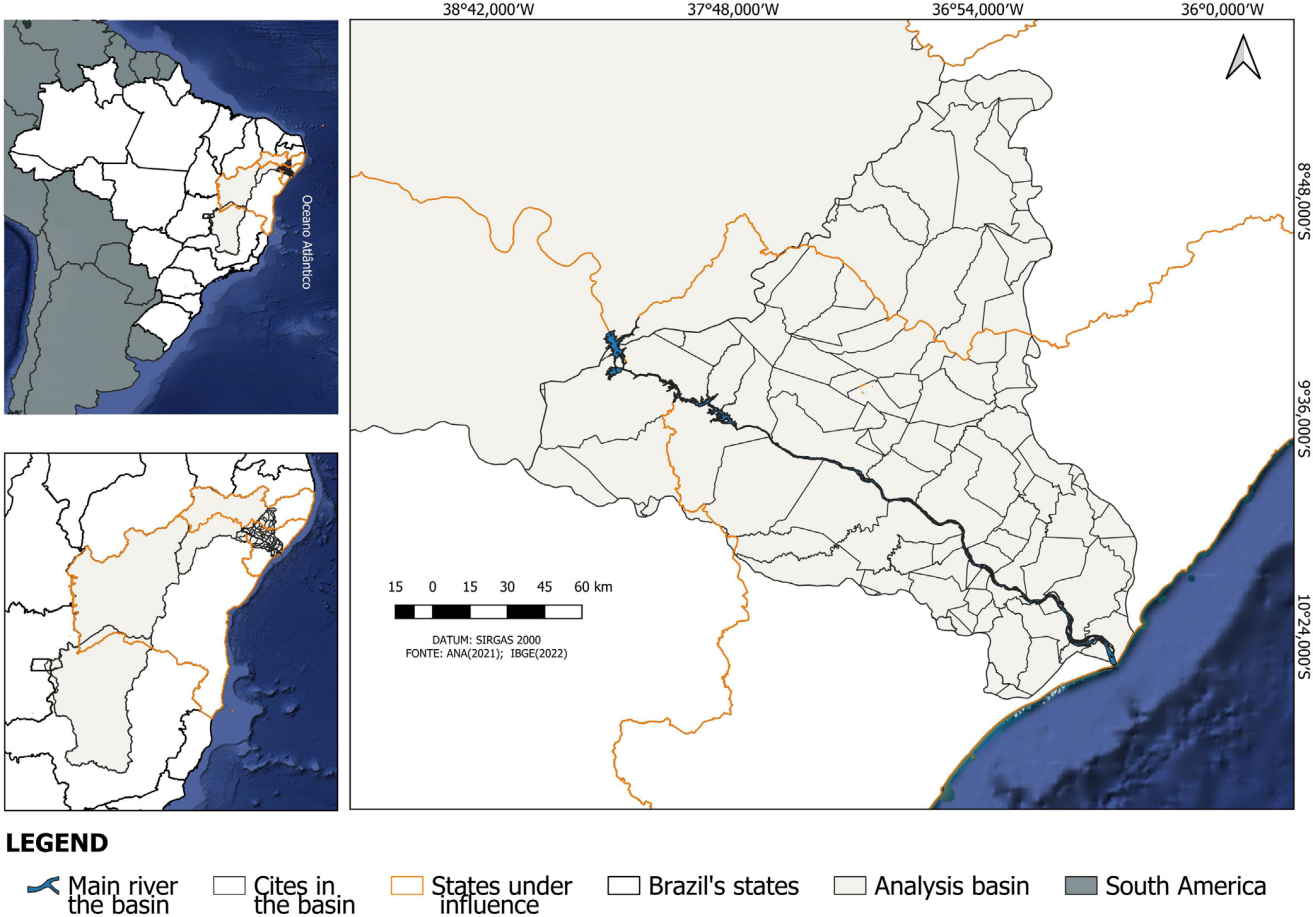
Location of the Lower São Francisco River Watershed (BHBSF), Brazil.

The main unit of analysis of this study is the Lower São Francisco River Watershed (BHBSF in Portuguese) (Figure 2). This watershed is one of the four climatic and physiographic regimes of the São Francisco River Watershed. Located in the Northeast Region of Brazil, the BHBSF covers a total area of 2,987,300 ha, distributed across 104 municipalities and four states: Alagoas (48.08% of the BHBSF area), Sergipe (27.88%), Pernambuco (18.27%) and Bahia (5.77%).

With most of its extent under a semiarid climate (*Caatinga* biome), the BHBSF has undergone intense changes in its vegetation cover over the years, largely due to the expansion of agricultural and livestock activities. According to Silva et al. (2021) the main environmental problems of Lower São Francisco (BSF) can be grouped into three categories: (1) impacts caused by urbanization (deforestation and soil impermeabilization); (2) impacts associated with livestock production (extensive grazing, erosion, biodiversity loss); and (3) impacts related to crop production (erosion, fragmentation of forest remnants, loss of biodiversity).

### 2.2 Land Cover and Land Use Data

To identify and delimit the main land uses and land cover in the BHBSF, we used data from Collection 7 of the MapBiomas Brazil platform, released in 2022, with a spatial resolution of 30 × 30 m. This collection applies automatic pixel‐by‐pixel classification procedures to the best images from 2021 obtained by the Landsat satellite (OLI/TIRS sensor) (MapBiomas Project 2023).

For landscape analysis, we considered the classes and subclasses defined by MapBiomas, namely: (1) Forest (Savanna Formation, Forest Formation, Wooded Sandbank Vegetation and Mangrove); (2) Non‐Forest Natural Formation (Salt Flat, Herbaceous Sandbank Vegetation, Grassland Formation, Wetland, Rocky Outcrops, and Other Non‐Forest Formations); (3) Farming (Pasture, Sugarcane, Forest Plantation, Temporary and Perennial Crops and Mosaic of Uses); (4) Non‐Vegetated Area (Urban Area, Beach and Dunes); and (5) Water Bodies (Rivers, Aquaculture, Ocean and Lakes).

### 2.3 Configuration of Forest Remnant Patches

Landscape ecology indices were calculated from vector data from forest fragments. We considered patches larger than 1 ha, based on the forest concept of forest defined by the Brazilian Forest Service (2019) and FAO (2018). We analyze the area, core area and shape metrics, as they reflect the effective habitat size, the proportion available without edge effect, and the degree of geometric irregularity of the fragment (Lang and Blaschke 2009) (Table 1, Equations (1), (2), (3), (4), (5)).

**TABLE 1:** Metrics used to calculate the configurational fragility of forest remnants, with description and ecological relevance.

Metric	Description	Ecological relevance	Acronym	Analysis parameter
Area (ha)	Size of the forest patch	Larger patches tend to have greater resilience	CA	Class area
Core area (ha)	Interior area excluding 50 m buffer from edge	Indicates habitat quality and edge effect reduction	TCA	Total core area
Core area index	Proportion of core area relative to total area	Indicates habitat integrity and edge effect exposure	CAI	Core area
Number of patches	Count of discrete forest remnants	Reflects fragmentation degree	NP	Patch
Mean shape index	Ratio of perimeter to area, averaged	Reflects exposure to edge effects	MSI	Patch shape

The total area of the remnants was divided into three size classes: patches smaller than 5 ha (small), between 5 and 50 ha (medium) and larger than 50 ha (large). The Number of Patches (NP) represents the total number of patches in each class. For the Total Core Area and Core Area Index (TCA and CAI, respectively), we considered the effective portion of the fragment available to edge‐sensitive species. For this analysis, we adopted a 50 m edge.

(1) $$\text{AREA}=0.5\sum \left(x_{i+1}-x_{i}\right)\left(y_{i+1}+y_{i}\right)$$

(2) NP=n

(3) $$\mathrm{TCA}=\sum C_{i}$$

(4) $$\mathrm{CA}I_{i}=\frac{CA_{\text{core},i}}{CA_{i}}\times 100$$

(5) $$\text{SHAPE}=\frac{p}{2\sqrt{\pi \cdot a}}$$

where *x* and *y* = coordinates of the *i*‐th polygon vertex; *C*_*i*_ = core area of the *i*‐th patch; *p* = perimeter; *a* = patch area; *π* = constant pi.

The thresholds employed for each metric were defined to represent structural regimes associated with edge influence and the potential to retain core area, rather than equal‐sized intervals. Area classes were established as 1–1.4 ha, 1–5 ha, 5–50 ha, and > 50 ha. The lower threshold (5 ha) distinguishes very small remnants structurally likely to be dominated by edge effects under a 50 m edge buffer. The intermediate class (5–50 ha) represents a transitional structural condition, while fragments larger than 50 ha are more likely to maintain consistent core area and reduced edge‐to‐area ratios.

This classification framework follows general landscape ecological theory demonstrating non‐linear ecological responses to habitat area and disproportionate structural effects in smaller fragments (Fahrig 2003; Haddad et al. 2015). While the conceptual logic of distinguishing structural regimes is transferable across fragmented landscapes, the numerical thresholds adopted here are operational and context‐dependent, reflecting the edge depth considered and the size distribution of forest patches within the study area. They are therefore not intended as universal ecological limits but as regionally appropriate classifications.

The edge threshold is defined as the boundary for edge sensitivity in the biomes present within the watershed. As demonstrated in the works of Antongiovanni et al. (2018), Magnago et al. (2015), Harper et al. (2005), and Laurance et al. (2002), Despite the dearth of knowledge regarding edge effects in the Caatinga, this study adopted a threshold of 50 m as the edge width, representing an operational and conservative compromise. This value was selected in light of the scarcity of specific parameters for the biome, with the objective of ensuring prudence and precision in the analysis. Although edge effects in dry tropical forests may extend beyond this distance, this cut‐off is sufficient to capture significant ecological changes. For instance, Baronio et al. (2025) documented alterations in pollinator communities within the initial few meters of the periphery. In a similar vein, Carvajal‐Cogollo and Urbina‐Cardona (2015) discovered variations in biodiversity (herpetofauna) along the edge‐interior gradient. The selection of 50 m as the initial analysis limit is further substantiated by the intricate nature of the Caatinga, where recurrent anthropogenic disturbance frequently intersects with the conventional edge effect (Antongiovanni et al. 2020). This choice is both logical and pragmatic, as it provides a viable starting point for the examination of configurational fragility until forthcoming research enhances our comprehension of the true extent of these impacts.

The Shape Index (MSI) was also considered, which can range from a standard regular condition (compact/circular) to an irregular form (linear). In this study, a mean MSI value ≤ 1.4 was adopted as indicative of a regular‐shaped path. This limit is based on the observation that none of the analyzed spots exhibited an MSI equal to 1.0. This finding aligns with the classification proposed by Lang and Blaschke (2009), who defined patches with an MSI up to 1.4 as “relatively compact” due to their slight rectangular shape, representing the least irregular shapes in the examined dataset. Patches with an MSI value ≥ 1.5 were classified as irregular (Figure S1).

All calculations were performed using ArcGIS 10.3 (licensed to the Laboratório de Cartografia e Geografia of the Universidade Federal da Paraíba) with the help of the V‐LATE 2.0 extension (Vector‐based Landscape Analysis Tools Extension).

### 2.4 Configurational Fragility of Forest Remnants

**FIGURE 3:**
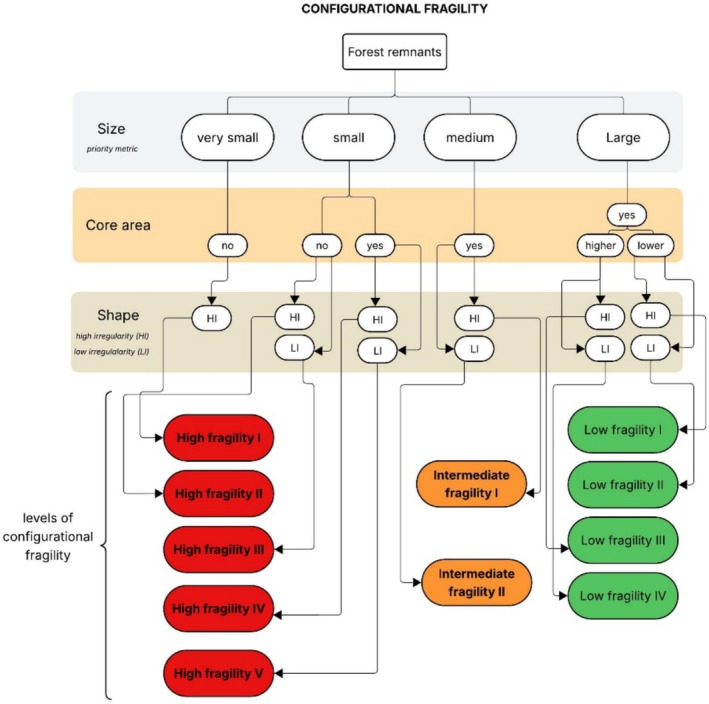
Decision framework for assigning levels of configurational fragility according to structural attributes of forest patches.

Configurational fragility was determined based on the interpretation of a set of configurational characteristics of forest remnants (Figure 3). For the interpretation of fragility, we follow the approach proposed by Lopes et al. (2022) and Matias et al. (2020) in which the values of patch‐level characteristics determine their fragility level (Table 2). Subsequently, the fragility sublevel was defined using qualitative metrics (Table 2).

**TABLE 2:** Parameters and levels of configurational fragility of forest remnants.

Configurational fragility level	Area	CAI	MSI	Observations
High fragility I	≥ 1 *e* < 1.4	0	≥ 1.5	Contains very small‐sized fragments
High fragility II	1.4 < *a* ≤ 5 ha	0	≥ 1.5	Contains very small‐sized fragments
High fragility III	1 < *a* ≤ 5 ha	0	< 1.5	Patches are more compact than other subclasses of high fragility
High fragility IV	1 < *a* ≤ 5 ha	> 0	≥ 1.5	Patches almost entirely affected by edge effect
High fragility V	1 < *a* ≤ 5 ha	> 0	< 1.5	Core area present; shapes more compact than other subclasses
Intermediate fragility I	5 < *a* ≤ 50 ha	> 0	≥ 1.5	More irregular patches compared to Intermediate II
Intermediate fragility II	5 < *a* ≤ 50 ha	> 0	< 1.5	More compact patches compared to Intermediate I
Low fragility I	≥ 50 ha	< 25%^a^	≥ 1.5	Irregular shapes among low fragility patches
Low fragility II	≥ 50 ha	< 25%^a^	< 1.5	More regular shapes among low fragility patches
Low fragility III	≥ 50 ha	≥ 25%	≥ 1.5	Favorable characteristics, but irregular shapes
Low fragility IV	≥ 50 ha	≥ 25%	< 1.5	Favorable characteristics of patches

^a^ Categories nested under low fragility due to the influence of area.

In addition, we considered the heterogeneity of the BHBSF, based on the criteria established by Ribeiro et al. (2009b), dos Santos et al. (2023) and Turner (1990), who classify configurational characteristics as either unfavorable or beneficial at the patch level. Therefore, the fragility levels were classified using the area (CA), the core area index (CAI) and the mean shape index (MSI), in priority order (Figure 3).

The determination of configurational fragility levels was carried out through a conditional selection of CA, CAI, and MSI metrics in a GIS environment, using qualitative–quantitative parameters that ranged from the worst to the best‐case scenarios of configurational fragility. From this conditional classification in the GIS environment, vector data were generated according to the sublevels of configurational fragility. This procedure allowed us to verify the land‐use context in which each level of configurational fragility is located.

The sublevels were classified according to the presence or absence of configurational characteristics (conditioning parameters) and classified as high, intermediate, or low fragility, varying from sublevel I to V (Figure 3, Table 2). These sublevels were necessary to encompass the diversity of configurational characteristics of the analyzed patches while simultaneously expressing the levels of compatibility of these fragments with favorable or unfavorable landscape configurations.

It is imperative to acknowledge that regions designated as exhibiting “low configurational fragility” may nevertheless be experiencing ecological degradation, as the configurational fragility examined in this study does not equate to ecological quality, a relationship that necessitates empirical validation.

### 2.5 Statistical Analysis

To ensure area standardization, we divided the BHBSF into 9 km^2^ grids (3 × 3 km). This consistency in size is crucial for spatial comparisons and subsequent statistical analyses. The choice of grid size was guided by conceptual and operational criteria, aiming to balance landscape heterogeneity with statistical robustness. A 3 × 3 km grid provides stable estimates of land‐use proportions from 30 m resolution data, reduces the occurrence of empty cells in semi‐arid mosaics, and represents landscape‐context processes rather than fine‐scale patch variability (see Figure S2).

Subsequently, we applied a distance‐based redundancy analysis (db‐RDA) using the *vegan* package in R (Legendre and Anderson 1999; Oksanen et al. 2001) to explore the relationship between the configurational fragility of forest fragments and the different types of land cover and land use in the BHBSF. The response variable was the configurational fragility matrix, which consists of the percentage cover of fragility levels of fragments in each grid. This matrix was transformed using the Hellinger method (Legendre and Gallagher 2001), which is suitable for compositional data with many zero values, fitting in our case. The transformation reduces the weight of extreme values and, more importantly, allows the appropriate use of the Euclidean distance, since it preserves the Euclidean geometry of the data. This procedure avoids distortions commonly associated with dissimilarity measures, such as Bray–Curtis, which can overestimate differences when there is a high occurrence of zeros.

As explanatory variables, we used the land cover and land use matrix, composed of the proportion of classes in each grid. These variables were centered and standardized (mean = 0, standard deviation = 1) to allow comparability on different scales. As spearman correlation analysis revealed marked negative correlations between natural vegetation and anthropogenic land uses, as well as strong positive correlations among coastal and wetland environments (Figure S3), we applied a principal component analysis (PCA) to reduce multicollinearity and improve ecological interpretability prior to db‐RDA. PCA was used to summarize the original classes into a reduced set of orthogonal components representing dominant land‐use gradients across the study area. The statistical significance of the global model and of individual land cover/use vectors was tested using 999 permutations, with the F statistic obtained from variance partitioning in distance space. This type of analysis has proven to be a reliable choice for complex datasets that do not follow normality assumptions (Jupke and Schäfer 2020).

## 3 Results

### 3.1 Land Cover and Land Use in a Watershed Under Anthropic Influence

The BHBSF is characterized by an anthropic matrix, with the Agriculture and Pasture class occupying 1,978,077 ha (66.92% of the watershed). Within this class, Pasture was the most representative subcategory, covering 57.75% of the watershed. In addition, 269,804 ha (9.13%) were classified as sugarcane monoculture and mosaic of use, concentrated mainly in the Atlantic Forest domain near the mouth of the São Francisco River. These productive activities are predominant in flat or gently undulating areas.

Only 30.08% of the BHBSF is covered by natural vegetation. The most representative subclass was the Savanna Formation, occupying 835,999.83 ha (28%), distributed irregularly in small, discontinuous patches. Other subclasses of natural vegetation included mangrove, wooded sandbank vegetation, herbaceous sandbank vegetation and forest formation, each occupying less than 1% of the watershed area.

### 3.2 Spatial Configuration of Forest Fragments

The BHBSF forest classes are divided into 37,022 forest patches, of which 26,700 were classified as small, 8856 as medium, and 1406 as large, distributed discontinuously over a total area of 835,563 ha. More than 72% of forest fragments in the watershed are considered small (< 5 ha). The relationship between the number of forest fragments in the BHBSF and the total area they occupy was inverse. Most patches were small in size; however, the sum of their total area was lower than that of medium and large fragments combined.

Regarding the analysis of core area (TCA), 41.8% (15,508 patches) had a null core area, indicating structures entirely under edge influence. Most of the forest fragments without core area were surrounded by pasture and/or sugarcane, especially those belonging to the Forest Formation subclass. The mean shape index (MSI) of the forest fragments was highly heterogeneous and varied according to the size of the patch. The small patches exhibited more regular shapes (average MSI = 1.45), whereas the medium and large patches showed more irregular shapes (average MSI = 2.35). Some fragments with MSI > 5 were larger than 50 ha and located close to watercourses, mainly along riparian forests of the main channel of the São Francisco River.

### 3.3 Configurational Fragility of Forest Remains

**FIGURE 4:**
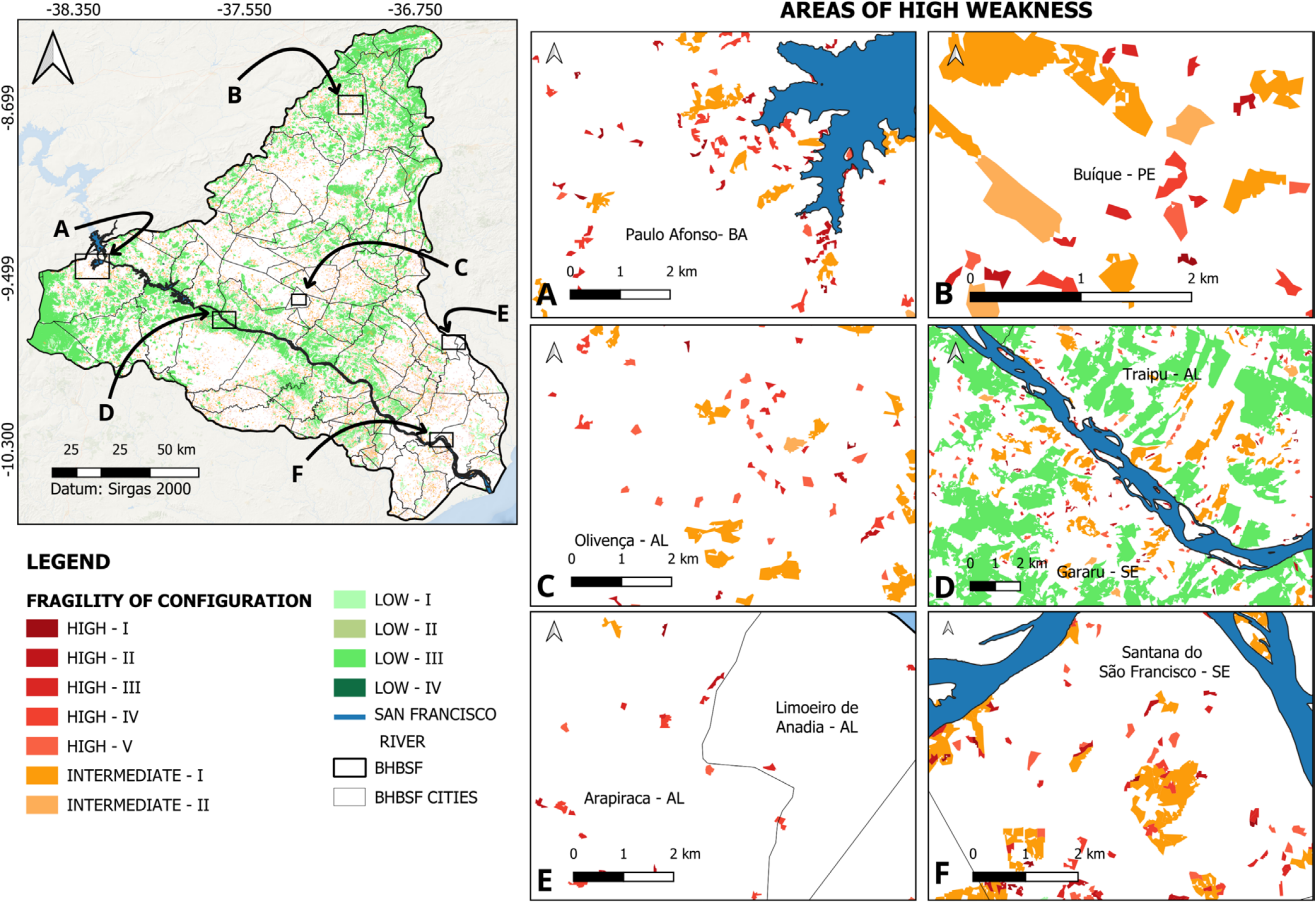
Configurational fragility of the forest structure in the Lower São Francisco River Watershed (BHBSF), showing the spatial distribution of fragility levels. AL, Alagoas; BA, Bahia state; PE, Pernambuco; SE, Sergipe. Sample areas of high fragility throughout the watershed: (A) Paulo Afonso, BA; (B) Buíque, PE; (C) Olivença, AL; (D) Traipu, AL and Gararu, SE; (E) Arapiraca, AL; (F) Santana do São Francisco, SE.

The levels of structural fragility in the forest patches ranged from high to low, with sublevels I to V. In particular, 72.2% of the patches exhibited high configurational fragility (Figure 4). The most fragile subclass was the Savanna Formation, with 96.1% of the patches classified within intermediate to high fragility levels, focusing on configurational deterioration and the degradation of the Caatinga biome in the watershed.

There was a predominance of patches with high levels of configurational fragility in all forest formations, with High Fragility III being the most representative level, comprising 8610 patches entirely affected by edge effects and of small size.

The distribution of patches in intermediate fragility levels was discontinuous throughout the watershed. However, more frequent records were observed in areas surrounded by the agriculture and pasture matrix, particularly the pasture subclass. Intermediate Fragility I was recorded in 7290 patches (19.7%), of which only 313 had an area greater than 40 ha, indicating the predominance of smaller fragments in the landscape.

Low levels of structural fragility were recorded in only 1406 patches (3.82%), of which 1260 were classified as Low Fragility III (patches > 50 ha with irregular shapes). This result highlights that the largest forest fragments in the BHBSF exhibit irregular shapes. In particular, only eight fragments, all belonging to the savanna formation, had configurational characteristics that categorized them as Low Fragility IV (patches with favorable configurational features).

### 3.4 Relationship Between Land Cover and Land Use and Configurational Fragility of Forest Remnants

The decomposition of the principal components revealed three dominant gradients of land use and land cover in the BHBSF (see Tables [Link], [Link] for a complete set). PC1 (*F* = 3.5 and *p* = 0.01), associated with herbaceous formations, wooded sandbank vegetation and wetlands, highlighted the fragility of coastal environments and areas with limited tree cover. PC2 (*F* = 2.8 and *p* = 0.03), emphasized a contrast between the savanna formation (positive values) and the pasture (negative values), establishing a clear separation between the natural ecosystems and the areas converted to agriculture and livestock. PC3 (*F* = 2.3 and *p* = 0.04), was associated with a combination of forest formations, forest plantation and mosaic of uses, representing the configurational heterogeneity typical of fragmented landscapes. Together, these three axes synthesized ecologically relevant gradients, responsible for a robust separation among the units analyzed in the watershed.

**FIGURE 5:**
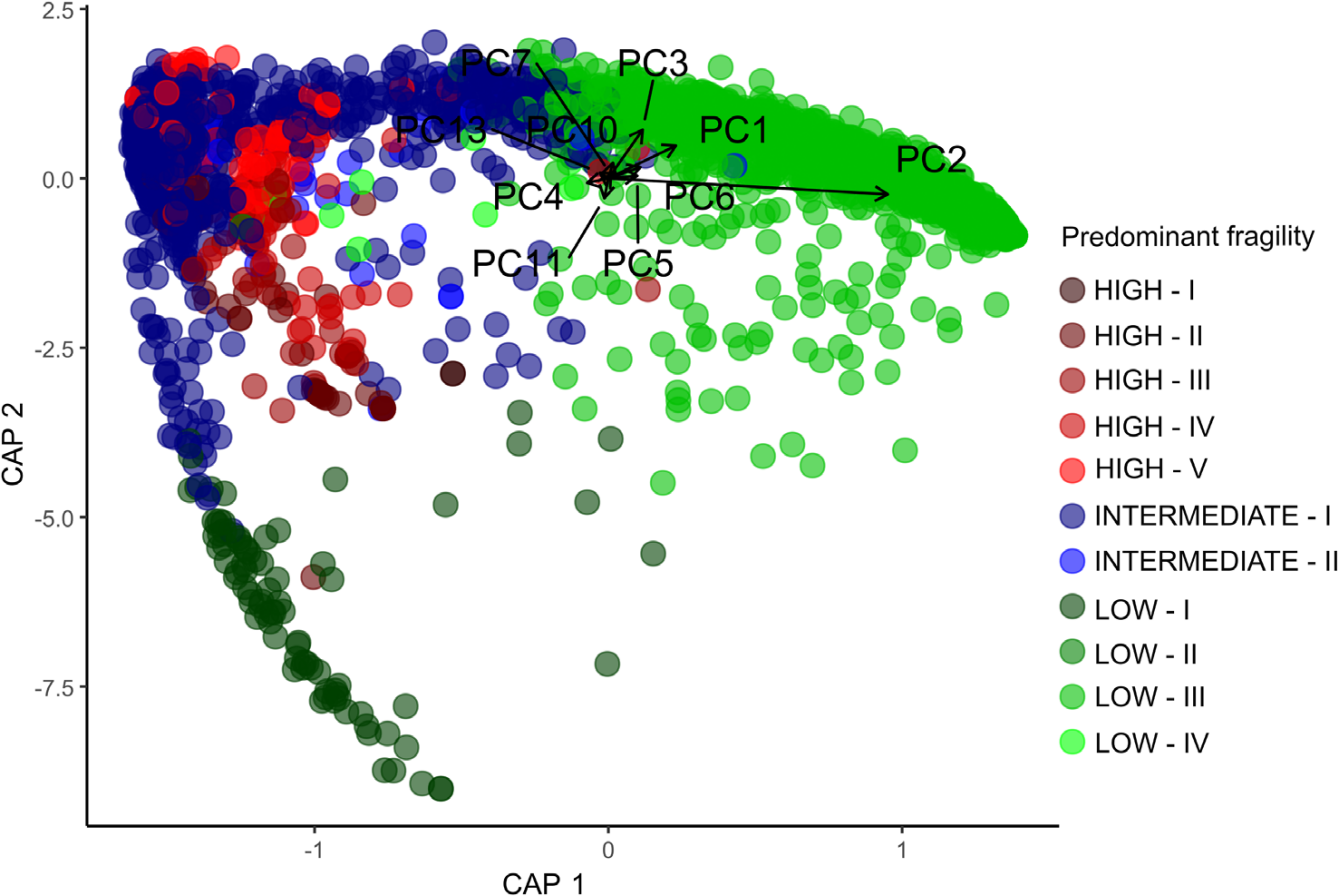
db‐RDA ordination showing the relationship between land cover/use and configurational fragility of forest fragments in the BHBSF. Legend: PC1—herbaceous formations, wooded sandbank vegetation and wetlands; PC2—contrast between savanna formation and pasture; PC3—forest formation, forest plantation and mosaic of uses; PC4—beaches and salt flat; PC5—rivers, lakes and ocean; PC6—sugarcane, non‐vegetated areas, and temporary crops; PC9—unknown class; PC10—non‐forest natural formations and mining; PC11—rocky outcrops; PC12—opposition between urban areas and perennial crops; PC13—aquaculture and mangroves.

The db‐RDA analysis was significant in explaining the variance in the configurational fragility data (adjusted *R* ^2^ = 0.362), which corresponds to 36.5% of the total variation. In particular, CAP1 accounted for 92.2% of the constrained variation, while CAP2 contributed 6.6%. Thus, although multiple gradients emerged in the PCA, the multivariate structure of the db‐RDA (Figure 5) indicated that most of the variation was explained by the main axis (CAP1), reflecting a clear opposition between natural environments and anthropogenic land uses.

## 4 Discussion

Our results provide an overall picture of the landscape configuration in a watershed under strong anthropogenic influence, characterized by small, discontinuous forest fragments that are largely affected by edge effects. We consistently demonstrate the influence of land use and land cover on the configurational fragility of natural forest fragments, both at the patch and landscape levels. On the grid scale (9 km^2^), the presence of Pasture was associated with a high configurational fragility of the forest fragments, while a greater coverage of natural forests (Savanna Formation and Forest Formation) was associated with a higher proportion of fragments classified as low configurational fragility. Importantly, this relationship does not imply a direct causal effect of forest cover per se, but rather reflects differences in the spatial organization of remnants, as areas with similar forest cover may exhibit contrasting fragment size distributions, shape complexity, and proportions of core area, which are explicitly captured by the configurational fragility index. These findings highlight the ecological relevance of land use dynamics for forest conservation and management in tropical and semi‐arid ecosystems, where agriculture and livestock production exert intense pressures on the landscape.

Although the association between anthropogenic land uses and increased fragment vulnerability is expected, the contribution of the configurational fragility approach lies not in demonstrating that agriculture fragments forests, but in qualifying how fragments are structurally organized under different landscape matrices. Unlike analyses based solely on patch size, the proposed index integrates area, shape and core area metrics in a hierarchical framework, allowing fragments of similar size to be distinguished according to their effective habitat availability and exposure to edge effects.

This distinction is particularly relevant for the intermediate fragility classes, which represent transitional structural conditions that are not captured by simple fragmentation metrics. In these cases, fragments embedded in heterogeneous agricultural mosaics may retain core areas and relatively compact shapes, whereas fragments of comparable size surrounded by homogeneous matrices tend to exhibit higher edge exposure and reduced functional habitat. Therefore, the fragility index provides additional ecological nuance by identifying priority areas where restoration or management actions may prevent the transition from intermediate to high configurational fragility—information that would not be evident from patch‐size maps alone.

Even though the classification relies on predefined thresholds, the predominance of high configurational fragility in the watershed reflects the marked structural condition of the landscape, characterized by a large number of small and edge‐dominated fragments. Thus, the observed pattern should be interpreted as a property of the spatial configuration itself rather than as a consequence of specific cut‐off values.

### 4.1 Landscape Panorama in the Watershed

The configurational aspects of forest patches in the BHBSF, including their discontinuous (fragmented) distribution and the predominance of small patches, clearly indicate watershed degradation. This is particularly relevant given the complexity of maintaining forests in tropical and semi‐arid environments under scarce rainfall regimes, coupled with intensive and extensive anthropogenic land use.

Several studies, such as Farias et al. (2023) and Qiu and Turner (2015), have identified in agro‐pastoral watersheds a reshaping of forest patches into increasingly discontinuous and smaller fragments. These configurational characteristics drive the loss of environmental quality of the landscape, expressed through progressive reductions in vegetation cover and biodiversity, the introduction of exotic species, the intensification of erosive processes, and the decline in the quality of ecosystem services (Antongiovanni et al. 2018; Bastos et al. 2023).

It should be noted that the small patches analyzed here are 67% below the global average size for tropical forest fragments (Taubert et al. 2018). This pattern is linked to a historical process of forest fragmentation, often associated with anthropogenic pressures. Although small forest patches are frequently associated with low diversity and limited habitat complexity (Fletcher et al. 2018), their functional importance should not be overlooked. Small patches can serve as stepping stones, temporary shelters and sources of propagules and seeds essential for the success of tree assemblages (Arasa‐Gisbert et al. 2021; Fahrig et al. 2019).

In agricultural matrices, small fragments can provide resources for pollinators and natural enemies of crop pests, act as physical barriers against wind and floods, and contribute to maintaining water quality and availability (Decocq et al. 2016). Thus, the lack of conservation or outright removal of these fragments may generate both environmental and socioeconomic losses (Riva et al. 2024).

### 4.2 Configurational Fragility and the Influence of Land Cover and Land Use

Despite the evident relationship between Pasture and high and intermediate configurational fragility (Figure 5), we also found that anthropogenic uses such as sugarcane plantations and mosaic of Uses contribute to increased levels of high and intermediate fragility. On the contrary, the Savanna Formation and Forest Formation tended to mitigate the levels of structural fragility (Table S1). This finding corroborates studies such as Ferrante et al. (2017) and Walder et al. (2025), which identified land use and occupation as major drivers of landscape structure. The expansion of pastures is often conditioned by the removal and/or fragmentation of native vegetation, thereby increasing structural fragility of the landscape (Caballero et al. 2023; Potapov et al. 2021).

Patches with intermediate and low configurational fragility were predominantly irregular in shape and exhibited low percentages of core area. This configuration was mainly associated with the presence of water bodies, whose landscape modifications are likely related to the high demand for water for irrigation or livestock watering. However, although these fragments were classified here as having low configurational fragility, their forest quality in terms of structure and species composition was not evaluated. Consequently, these patches may be under a greater degree of ecological degradation than indicated solely by their configurational characteristics.

Another factor to consider is that, possibly due to limitations inherent to remote‐sensing‐based land‐cover classification, some large patches classified as Low Fragility III (> 50 ha) along the riparian areas of the São Francisco River (Figure 3) may not reflect actual field conditions. Regional field studies (Almeida et al. 2024; Silva et al. 2021), as well as observations by the authors, indicate that these apparently continuous matrices may harbor microscale fragmentation (e.g., degradation, trails, clearings) that the sensor cannot distinguish. This caveat underscores the notion that the configurational fragility identified in this study functions on a landscape scale and may, in higher‐resolution analyses, prove to be even more pronounced than that detected by the sensor utilized.

In addition, the present analysis represents a static snapshot of landscape configuration based on land‐use and land‐cover data from 2021. Temporal dynamics, such as changes in fragmentation patterns and land‐use trajectories over time, were not explicitly evaluated and could be addressed in future studies using multi‐temporal approaches.

Finally, because the statistical analyses were conducted using contiguous spatial grids, some degree of spatial autocorrelation among neighboring units is expected. Although the db‐RDA approach is appropriate for exploring multivariate associations between land‐use gradients and configurational fragility, spatial dependence was not explicitly modeled. Therefore, the results should be interpreted as spatially structured associations rather than strictly independent effects, and future studies could incorporate spatially explicit methods to disentangle environmental and spatial componentes.

## 5 Conservation Insights

It is important to acknowledge that this study has certain limitations: (i) MapBiomas use can underestimate degradation on the microscale, failing to capture the structural reality of small forest fragments; (ii) variables such as land use history, floristic composition and the presence of key species were not assessed, although they may alter interpretations of fragility; and (iii) the 9 km^2^ grid resolution is suitable for regional patterns but can mask fine‐scale processes relevant for species with low mobility or restricted home ranges. Thus, the results should be interpreted as general trends, useful for guiding macro‐scale policies, but requiring complementary local studies.

Despite these limitations, our findings point to strong conservation implications. The spatial patterns identified in the watershed indicate that high configurational fragility is concentrated in landscape contexts dominated by pasture and agriculture, where small fragments and absence of core area are prevalent. Given the high proportion of fragments under high fragility (72.2%), strategic ecological restoration should urgently prioritize increasing the area and connectivity of small fragments, particularly in landscape contexts dominated by intensive land use, in order to reduce edge effects and expand core areas.

In addition, fragments located near water bodies deserve particular attention, as our results showed that larger riparian patches often exhibit irregular shapes and reduced core areas, despite their larger total area. In parallel, the implementation of ecological corridors among larger forest fragments may enhance functional connectivity, favoring dispersal and pollination flows.

The prevalence of pasture (57.7%) associated with high fragility justifies the adoption of silvopastoral and agroforestry systems to buffer impacts on fragments surrounded by pasture, as well as the establishment of buffer zones with native vegetation in areas of monoculture and extensive livestock production. These measures are especially relevant in areas where intermediate and high fragility classes were most frequent, indicating priority zones for restoration and management actions.

Ultimately, effective conservation in the watershed requires coordinated actions across multiple scales, from farm‐level practices to regional land use policies, supported by scientific data and continuous monitoring of landscape fragility.

The configurational fragility does not inherently lead to ecological degradation. Analyses that integrate effective data are needed to identify levels of degradation, such as data related to the composition and functionality of forest fragments, which were not measured in this study.

Future research should investigate how configurational fragility affects fundamental ecological processes such as pollination, seed dispersal, and persistence of key species, especially in fragments without core areas. Another important direction is to integrate socioeconomic dimensions, evaluating how fragility influences ecosystem services (e.g., water provision, climate regulation, erosion control) and impacts the sustainability of rural communities in the watershed.

## 6 Conclusions

The results of this study confirm the initial hypothesis that the process of landscape anthropization is a driving force behind the configurational fragility of forest remnants. However, the present findings extend beyond the observation that anthropized landscapes predominantly feature small fragments. The present study operationalizes configurational fragility at hierarchical sublevels, thereby demonstrating that landscapes exhibiting similar proportions of forest cover can exhibit substantially different degrees of configurational fragility, depending on how the remnants are spatially organized within the matrix.

In BHBSF, anthropization manifested itself not only through the widespread occurrence of patches < 5 ha, but also through a structured spatial reorganization, expressed by the predominance of specific sublevels of fragility, especially High III and Intermediate I, with occasional occurrences of Low III. This pattern reveals a landscape in which small fragments strongly dominated by edges coexist with relatively larger remnants, but with irregular geometries that increase exposure to edges and structural vulnerability.

From an applied perspective, three priority areas are highlighted: (i) protection of remnants classified as Low III, which can act as strategic structural nuclei; (ii) preventive interventions in Intermediate I fragments, focusing on reducing edge effects, increasing local connectivity, and improving landscape flow; and (iii) configurational restoration in High III areas, aiming to mitigate excessive exposure to the edge and favor the retention of ecological processes.

Furthermore, the results of this study indicate that landscapes with similar levels of forest cover may differ substantially in their configurational fragility depending on the spatial organization of the fragments. The observed association between greater native forest cover and lower fragility may potentially reflect differences in spatial configuration and matrix permeability, rather than being a direct causal effect of the amount of forest itself.

In order to further refine our comprehension of the interplay between spatial configuration, ecological processes, and structural quality, subsequent studies ought to integrate sublevels of fragility into functional indicators (e.g., functional connectivity, presence of sensitive guilds, or maintenance of ecosystem services). Additionally, incorporating temporal dynamics into the research design would facilitate a more nuanced examination of the relationship between spatial configuration and ecological processes.

## Author Contributions

**Jessyca Janyny de Oliveira Saraiva‐Maia:** conceptualization (supporting), data curation (lead), formal analysis (equal), investigation (equal), methodology (equal), resources (equal), writing – original draft (lead), writing – review and editing (equal). **Milena Dutra da Silva:** conceptualization (lead), data curation (supporting), formal analysis (equal), investigation (supporting), methodology (equal), resources (equal), writing – original draft (supporting), writing – review and editing (equal). **Wallace Beiroz:** formal analysis (equal), investigation (supporting), methodology (supporting), resources (equal), writing – review and editing (equal). **Nadjacleia Vilar Almeida:** conceptualization (lead), data curation (supporting), formal analysis (equal), investigation (lead), methodology (lead), resources (supporting), writing – original draft (lead), writing – review and editing (equal).

## Funding

This work was supported by Coordenação de Aperfeiçoamento de Pessoal de Nível Superior (CAPES).

## Conflicts of Interest

The authors declare no conflicts of interest.

## Supporting information


**Figure S1:** Distribution of structural metrics used to define configurational fragility. (A) Patch area (log10 scale); (B) Mean Shape Index (MSI) with threshold at 1.5; (C) Core Area Index (CAI, %) with reference at 25%; (D) Variation of MSI across area classes.

**Figure S2:** Histogram of the sampling grid. The 3 × 3 km resolution was selected because it provides robust estimates of land use proportions (based on 30 m data). The grid adopted adequately represents landscape context processes, reducing fine‐scale variability and cells without data, according to operational and conceptual criteria.

**Figure S3:** Spearman correlation matrix among land‐use and land‐cover (LULC) classes. Strong correlations were observed among several classes, particularly between natural vegetation and anthropogenic uses, as well as among coastal and wetland environments, indicating high multicollinearity and supporting the use of principal component analysis (PCA) to derive independent land‐use gradients prior to db‐RDA.

**Table S1:** Contribution of land‐cover and land‐use classes to principal components (PCs) used in the db‐RDA analysis.

**Table S2:** Eigenvalues and explained variance of db‐RDA axes for configurational fragility in the Lower São Francisco River Basin.

**Table S3:** Results of the permutation test (ANOVA by terms) for the db‐RDA model, showing the variance explained, *F*‐values, and significance (*p*‐value) for each predictor variable.

## Data Availability

Data supporting the results of this study are openly available in Dataverse DATAPB: https://doi.org/10.71650/DATAPB/AUBIHO (Saraiva‐Maia et al. 2026).
