# Supplementary figures and images for: Configurational Fragility of Forest Landscapes Under Multiple Anthropic Uses
Source: Ecol Evol. 2026 Jun 11;16(6):e73460. doi: 10.1002/ece3.73460 (PMC13259973; doi:10.1002/ece3.73460)

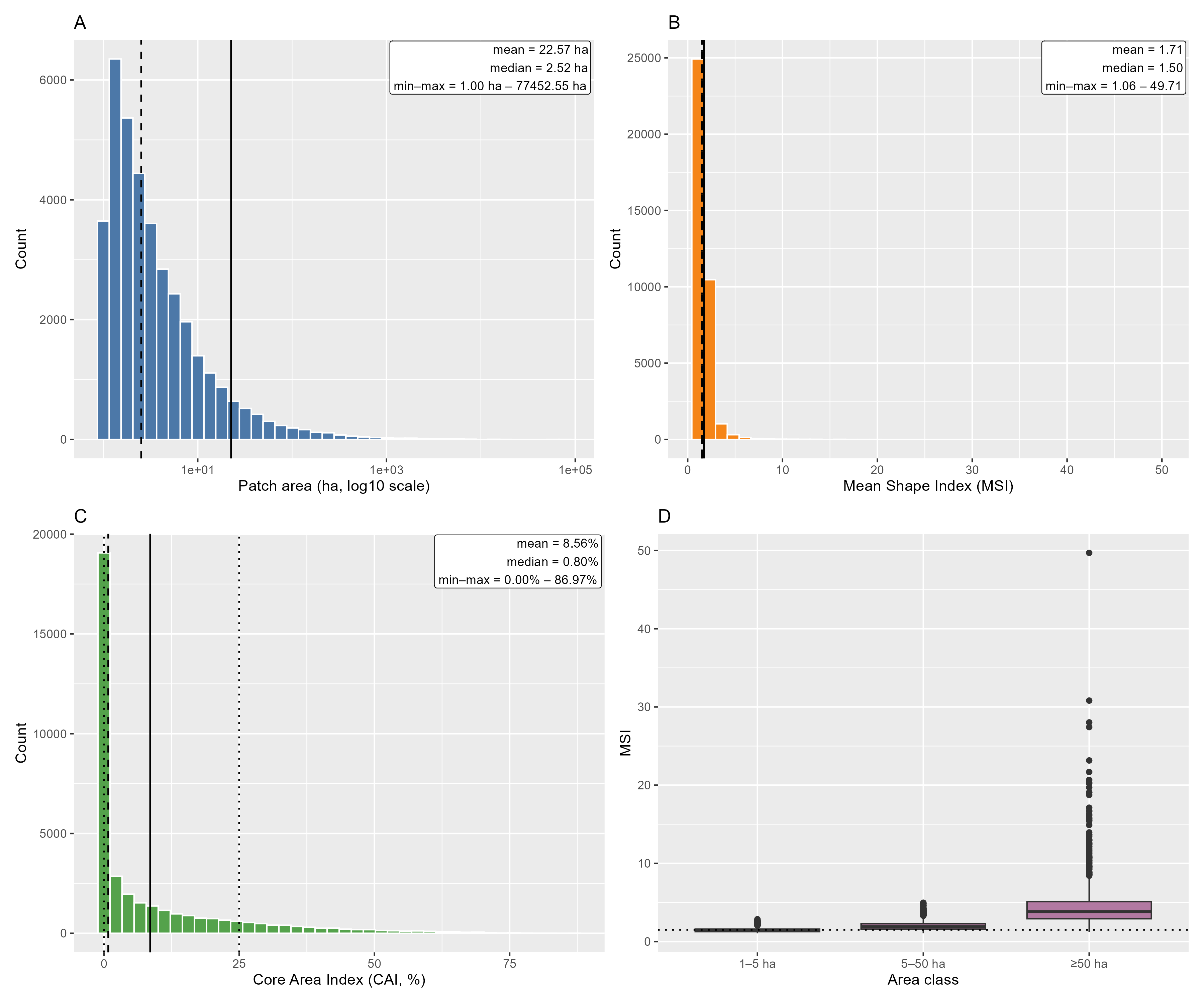

Supplement: Supplementary file 1 — Figure S1: Distribution of structural metrics used to define configurational fragility. (A) Patch area (log10 scale); (B) Mean Shape Index (MSI) with threshold at 1.5; (C) Core Area Index (CAI, %) with reference at 25%; (D) Variation of MSI across area classes. [file ECE3-16-e73460-s004.png]

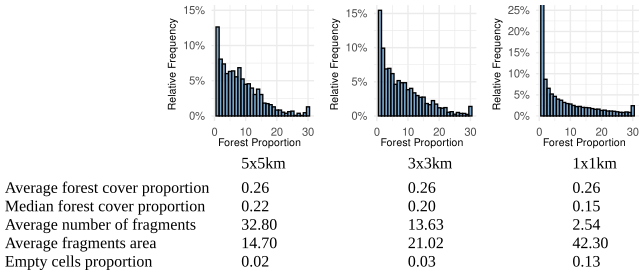

Supplement: Supplementary file 2 — Figure S2: Histogram of the sampling grid. The 3 × 3 km resolution was selected because it provides robust estimates of land use proportions (based on 30 m data). The grid adopted adequately represents landscape context processes, reducing fine‐scale variability and cells without data, according to operational and conceptual criteria. [file ECE3-16-e73460-s003.png]

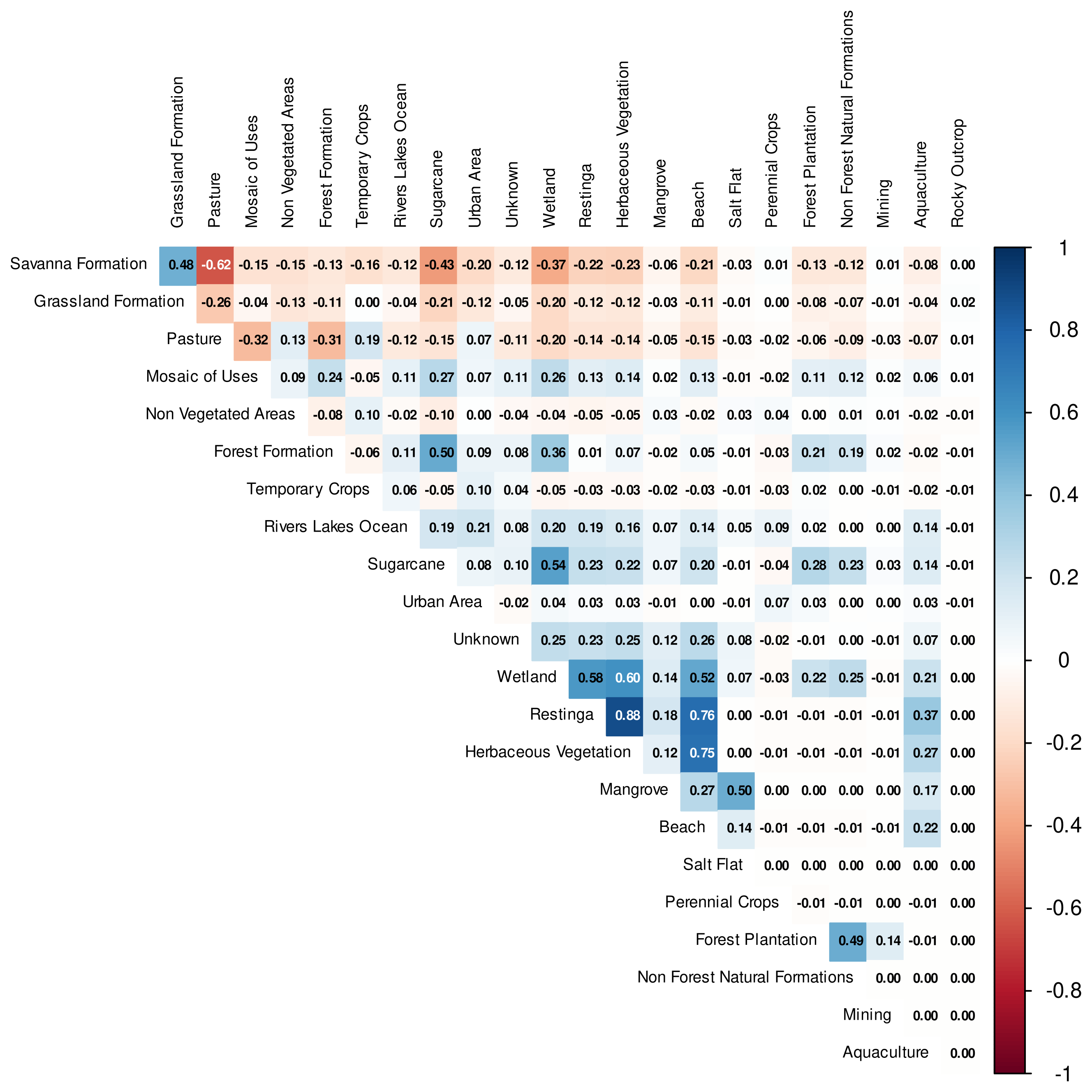

Supplement: Supplementary file 3 — Figure S3: Spearman correlation matrix among land‐use and land‐cover (LULC) classes. Strong correlations were observed among several classes, particularly between natural vegetation and anthropogenic uses, as well as among coastal and wetland environments, indicating high multicollinearity and supporting the use of principal component analysis (PCA) to derive independent land‐use gradients prior to db‐RDA. [file ECE3-16-e73460-s005.png]
