# Supplementary material for: Configurational Fragility of Forest Landscapes Under Multiple Anthropic Uses
Source: Ecol Evol. 2026 Jun 11;16(6):e73460. doi: 10.1002/ece3.73460 (PMC13259973; doi:10.1002/ece3.73460)
Supplement: Supplementary file 4 — Table S1: Contribution of land‐cover and land‐use classes to principal components (PCs) used in the db‐RDA analysis. [file ECE3-16-e73460-s002.docx]

**Table S1**. Contribution of land-cover and land-use classes to principal components (PCs) used in the db-RDA analysis.

| **Land-cover/land-use class** | **PC** | **Value** |
| --- | --- | --- |
| Herbaceous Formation | PC1 | -0.4025 |
| Wooded Sandbank Vegetation | PC1 | -0.4067 |
| Wetland | PC1 | -0.4725 |
| Grassland Formation | PC2 | 0.3711 |
| Pasture | PC2 | -0.5849 |
| Savanna Formation | PC2 | 0.6704 |
| Silviculture | PC3 | -0.3362 |
| Mosaic of Uses | PC3 | -0.3379 |
| Forest Formation | PC3 | -0.4516 |
| Beach | PC4 | 0.4795 |
| Salt Flat — saline plain | PC4 | 0.6488 |
| Rivers, Lakes, and Ocean | PC5 | -0.4538 |
| Sugarcane | PC6 | -0.3580 |
| Non-vegetated Areas | PC6 | 0.4991 |
| Temporary Crops | PC6 | 0.5403 |
| Unknown | PC9 | 0.8603 |
| Non-forest Natural Formations | PC10 | 0.4417 |
| Mining | PC10 | -0.5660 |
| Rocky Outcrops | PC11 | 0.7734 |
| Urban Area | PC12 | 0.5640 |
| Perennial Crops | PC12 | -0.7767 |
| Aquaculture | PC13 | 0.5168 |
| Mangrove | PC13 | -0.7655 |

*Note: Within each principal component (PC), variables are ordered by increasing contribution (absolute values).*
