# Supplementary material for: Configurational Fragility of Forest Landscapes Under Multiple Anthropic Uses
Source: Ecol Evol. 2026 Jun 11;16(6):e73460. doi: 10.1002/ece3.73460 (PMC13259973; doi:10.1002/ece3.73460)
Supplement: Supplementary file 5 — Table S2: Eigenvalues and explained variance of db‐RDA axes for configurational fragility in the Lower São Francisco River Basin. [file ECE3-16-e73460-s001.docx]

**Table S2***.* Eigenvalues and explained variance of db-RDA axes for configurational fragility in the Lower São Francisco River Basin.

| Axis | Eigenvalue | Proportion | Cumulative |
| --- | --- | --- | --- |
| CAP1 | 462,9717 | 0,9221 | 0,9221 |
| CAP2 | 33,374 | 0,06647 | 0,98857 |
| CAP3 | 2,982456 | 0,00594 | 0,99451 |
| CAP4 | 1,644148 | 0,003275 | 0,997789 |
| CAP5 | 0,706844 | 0,001408 | 0,999197 |
| CAP6 | 0,3315851 | 0,0006604 | 0,9998574 |
| CAP7 | 0,03027 | 0,00006029 | 0,999918 |
| CAP8 | 0,02751 | 0,00005479 | 0,999973 |
| CAP9 | 0,01078 | 0,00002146 | 0,999995 |
| CAP10 | 0,002955 | 0,000005885 | 1 |
| CAP11 | 0,00006427 | 0,000000128 | 1 |
