# Supplementary material for: Configurational Fragility of Forest Landscapes Under Multiple Anthropic Uses
Source: Ecol Evol. 2026 Jun 11;16(6):e73460. doi: 10.1002/ece3.73460 (PMC13259973; doi:10.1002/ece3.73460)
Supplement: Supplementary file 6 — Table S3: Results of the permutation test (ANOVA by terms) for the db‐RDA model, showing the variance explained, F‐values, and significance (p‐value) for each predictor variable. [file ECE3-16-e73460-s006.docx]

**Table S3***.* Results of the permutation test (ANOVA by terms) for the db-RDA model, showing the variance explained, F-values, and significance (p-value) for each predictor variable.

| Variable | Df | Variance | F_value | p-value |
| --- | --- | --- | --- | --- |
| PC1 | 1 | 50 | 3,5 | 0,01 |
| PC2 | 1 | 45 | 2,8 | 0,03 |
| PC3 | 1 | 38 | 2,3 | 0,04 |
| PC4 | 1 | 30 | 2,1 | 0,05 |
| PC5 | 1 | 25 | 1,9 | 0,06 |
| PC6 | 1 | 20 | 1,7 | 0,08 |
| PC7 | 1 | 18 | 1,6 | 0,09 |
| PC8 | 1 | 15 | 1,5 | 0,1 |
| PC9 | 1 | 12 | 1,4 | 0,12 |
| PC10 | 1 | 10 | 1,3 | 0,15 |
| PC11 | 1 | 8 | 1,2 | 0,18 |
| PC12 | 1 | 7 | 1,1 | 0,2 |
| PC13 | 1 | 6 | 1 | 0,25 |
| Residual |  |  |  |  |
